# First record of *in vitro* formation of ectomycorrhizae in *Psidium cattleianum* Sabine, a native Myrtaceae of the Brazilian Atlantic Forest

**DOI:** 10.1371/journal.pone.0196984

**Published:** 2018-05-08

**Authors:** Cassio Geremia Freire, Admir José Giachini, João Peterson Pereira Gardin, Ana Claudia Rodrigues, Renato Luis Vieira, César Milton Baratto, Simone Silmara Werner, Bernardo Haas Abreu

**Affiliations:** 1 Departament of Health and Biology Sciences, University Alto Vale do Rio do Peixe, Caçador, Santa Catarina, Brazil; 2 Department of Microbiology, Immunology and Parasitology, Federal University of Santa Catarina, Florianópolis, Santa Catarina, Brazil; 3 Department of Plant Physiology, West University of Santa Catarina, Videira, Santa Catarina, Brazil; 4 Department of Fungi, Algae and Plants Biology, Federal University of Santa Catarina, Florianópolis, Santa Catarina, Brazil; 5 Department of Temperate Climate Plants, Agricultural Research and Rural Extension Company of Santa Catarina, Caçador, Santa Catarina, Brazil; 6 Departament of Molecular Biology, West University of Santa Catarina, Videira, Santa Catarina, Brazil; 7 Department of Mathematics and Statistics, Agricultural Research and Rural Extension Company of Santa Catarina, Lages, Santa Catarina, Brazil; 8 Department of Applied Microscopy, Federal University of Santa Catarina, Florianópolis, Santa Catarina–Brazil; Dong-A University, REPUBLIC OF KOREA

## Abstract

Like many other species of trees native to the Brazilian Mata Atlântica (Atlantic Forest), the Myrtaceae, such as the Red Araza (*Psidium cattleianum* Sabine), are widely cited as arbuscular mycorrhizal formers. Nevertheless, recent studies show evidence that Myrtaceae from different tropical, subtropical and neotropical ecosystems can also prompt the formation of ectomycorrhizae, indicating that this species' ectomycorrhizal status should be further explored. Because of this, this research effort studied the *in vitro* interaction between the Red Araza and two ectomycorrhizal fungi isolates, belonging to the *Pisolithus microcarpus* (D17) and *Scleroderma citrinum* (UFSC-Sc133) species. An analysis was performed to determine the formation of ectomycorrhizal structures, or lack thereof, and the developmental differences between the *in vitro* mycorrhized and non-mycorrhized plants. The analysis proved that indeed an ectomycorrhizal association was developed between the Red Araza, and the D17 and UFSC-Sc133 isolates, a fact never before registered in the existing literature. After an *in vitro* period of 110 days, it was confirmed that the D17 and UFSC-Sc133 isolates formed mycorrhizal colonization of 91.6% and 15.7%, respectively. Furthermore, both isolates also promoted root thickening, and the formation of a fungal mantle and a Hartig net. However, when compared to the Control plants, the fungal isolates did not contribute to an increase in the development of the subject plants, possibly due to the specific experimental conditions used, such as a high humidity environment and high availability of nutrients in the symbiotic substrate.

## Introduction

Ectomycorrhizal fungi (fEcM) are symbiont organisms that dwell in the root systems of plants, forming one of the most important and diversified mutualistic relationships in the planet [1]. It is estimated that approximately 10% of all tracheophyte plant species exhibit this type of symbiosis, a percentage that can reach up to 90% of all tree species in temperate regions, especially the ones belonging to the Pinaceae, Betulaceae, Fagaceae, Salicaceae, Dipterocarpaceae and Myrtaceae families [2].

Historically, ectomycorrhizal symbiosis was considered to be restricted to the world's temperate regions, such as the coniferous forests of the Northern Hemisphere, where many of the plants form ectomycorrhizae and depend strictly on this association [3]. Yet, many recent research efforts [4–8] and evidence accumulated through the years [9] indicate that the ectomycorrhizal plants and fungi are also present in different tropical, subtropical [10], and neotropical [11] ecosystems.

In the last 50 years, Latin America has been the site of many different research efforts involving ectomycorrhizal fungi in tropical forests. A rich diversity of ectomycorrhizal fungi was evidenced in connection with more than 180 species found in Brazil, Guyana, Venezuela, and Colombia [12]. The following are among the main fungal genera found: *Scleroderma*, *Russula*, *Amanita*, *Lactarius*, *Clavulina*, *Inocybe* and *Craterellus* [7]. Additionally, a few plant species found in the Guyanas were confirmed to be independent of ectomycorrhizal fungi, and are believed to be endemic to that neotropical region [5].

In Brazil, the presence of ectomycorrhizal fungi has already been observed in the Amazon Forest [4,13] and in plants native to the Brazilian savanna [14]. Recent studies have also reported the presence of certain species of *Scleroderma* associated with native Dune Forests trees in the Brazilian Northeast [6,15]. Such studies show that ectomycorrhizal fungi are an integral part of the biodiversity found in Brazilian tropical forests [8], and that it's irrefutable that these ectomycorrhizal relationships actively alter and transform life, and the countless diverse interactions that occur in those ecosystems [16].

Ectomycorrhizal relationships are well known and well-studied within the symbiont families of temperate climates. With the exception of the genus *Eucalyptus*, however, there is very little information available about species from the Myrtaceae family, specifically those from tropical ecosystems. If we consider that some Myrtaceae are quite dominant in the floristic composition of Brazilian biomes, such as ecosystems within the Atlantic Forest [17], it seems odd that the study of ectomycorrhizal interaction, as it relates to the Brazilian native Myrtaceae, has received very little attention, even though it may play a complex ecological role in those regions [18,19].

The Red Araza (*Psidium cattleianum* Sabine) is a Myrtaceae that can be found in all of the Brazilian territory [20], and in other countries in Central and South America [21]. The species displays characteristics of phytochemical [22,23], nutritional [24] and ecological [25,26] importance. Until the moment, studies have shown that the Red Araza can make symbiotic relationships with endomycorrhizal fungi, but not with ectomycorrhizal fungi. This also occurs for most of Myrtaceae to the Brazilian Atlantic Forest. Nevertheless, studies in other tropical and subtropical ecosystems of the world, have shown that many species of Myrtaceae have ectomycorrhizal formations [4,5,12], including some data for the northeast of Brazil [6,15]. This information suggests that the number of native Myrtaceae species capable of forming ectomycorrhizae may be underestimated in the Brazilian Atlantic Forest, possibly due to the difficulties of collection and analysis ectomycorrhizae in these natural environments [3,18].

According to the above, there is an obvious need for more research into the actual ectomycorrhizal *status* of Myrtaceae native to the Brazilian Atlantic Forest. The purpose of this study, therefore, was to verify the occurrence of an *in vitro* interaction between the Red Araza and ectomycorrhizal fungi, and assess whether the formation of a symbiotic relationship, or lack thereof, affected in any way the development of the plant. Fungi of genera *Pisolithus* and *Scleroderma* were chosen for this study because they already have demonstrated the ability to form associations with different Myrtaceae species in tropical, subtropical and neotropical ecosystems [7,8,11]. Therefore, the formation of *in vitro* ectomycorrhizae in Red Araza could suggest that this and other native Myrtaceae in Brazil can form the same interactions in natural environments.

## Material and methods

### Ectomycorrhizal fungi isolates

The isolates used in this experiment were obtained from the fEcM inventory belonging to the Federal University of Santa Catarina's Department of Microbiology, Immunology and Parasitology (MIP-UFSC).

The fungi used were: the D17 isolate–*Pisolithus microcarpus*, collected from *Corymbia* sp.—a segregated genus of *Eucalyptus*, Myrtaceae—plantations (in Diamantina, State of Minas Gerais, Brazil); and the UFSC-Sc133 isolate–*Scleroderma citrinum*, collected from *Pinus elliottii* plantations (in Florianópolis, State of Santa Catarina, Brazil). Axenic cultures of these fungi were cultivated in Petri dishes in MNM culture medium [27], modified and supplemented with micronutrients in order to obtain the following concentrations (in mg L^-1^): KH_2_PO_4_, 500; CaCl_2_.2H_2_O, 50; (NH_4_)_2_HPO_4_, 1000; MgSO_4_.7H_2_O, 200; FeCl_3_, 12; NaCl, 25; C_6_H_12_O_6_, 10000; C_12_H_22_O_11_, 5000; malt extract, 3000; Thiamine-HCl, 0.1; MnSO_4_.H_2_O, 33.8; ZnSO_4_.7H_2_O, 17.2; H_3_BO_3_, 12.4; Kl, 1.66; CuSO_4_.5H_2_O, 0.05; Na_2_MoO_4_.2H_2_O, 0.5; CoCl_2_.6H_2_O, 0.05. Note that 8 g L^-1^ of agar were added after pH level adjustments (5.8±0.05).

**Fig 1:**
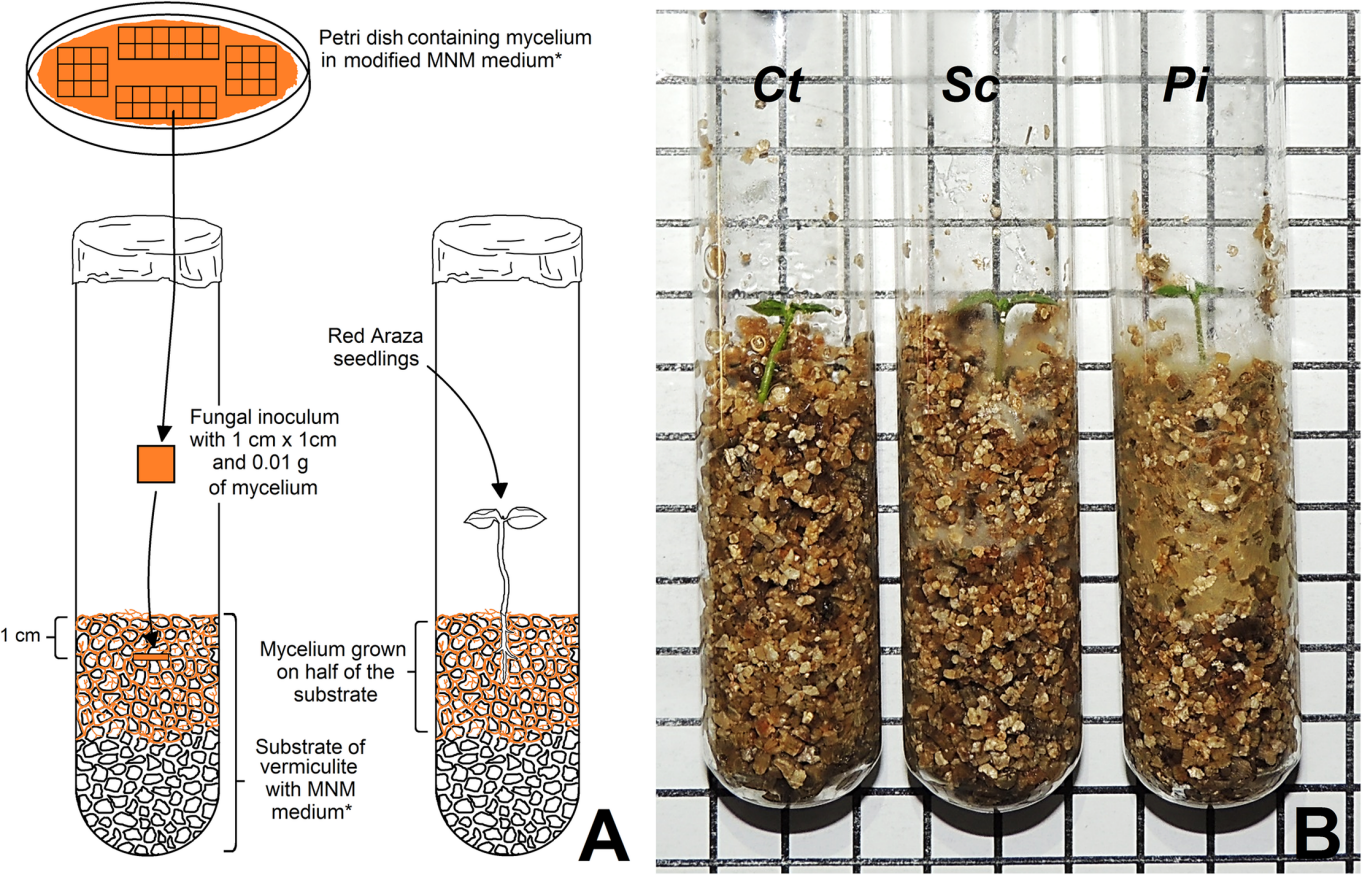
Schema representing the bioassay of the *in vitro* association between *Psidium cattleianum* (Red Araza) and ectomycorrhizal fungi D17 and UFSC-Sc133. (A) Methodology for the symbiosis bioassay. (B) Test tubes containing vermiculite and Red Araza seedlings, where: *Ct*, control (no fungi added); *Sc*, treated with mycelium from the UFSC-Sc133 (*Scleroderma citrinum*) isolate; and Pi, treated with mycelium from the D17 (*Pisolithus microcarpus*) isolate. *Modified Melin-Norkrans Medium [27] containing 10 g of glucose, 5 g of sucrose, and 200% of the micronutrients from an MS0 medium [28]. Squares were dimensioned at 1 cm by 1 cm.

Petri dishes remained in a BOD incubator, at 25±1°C, for 16 days, until mycelium growth was observed near the rims of the plates (Fig 1).

### Growing the Red Araza seedlings

Red Araza seeds were collected from fully ripened fruits taken from adult trees (at 26°49’06”S, 50°59’29”W) in the Experimental Field of Epagri of Caçador/SC (authorized by Dr. Renato Luis Vieira, research manager of Epagri—Agricultural Research and Rural Extension Company of Santa Catarina). The seeds were extracted using a sieve and running water, and dried at ambient temperature.

The dried seeds were disinfected in a laminar air flow cabinet with 70% v/v alcohol, for 1 minute, followed by a 20-minute immersion in sodium hypochlorite (NaClO, 1.5% active ingredient) with 6 drops L^-1^ of Tween® under constant agitation, and subsequently washed five times in distilled sterile water. Next, the seeds were germinated *in vitro* (flasks with 230 mL capacity) containing 55±2 grams of autoclaved sand (for 20 minutes, at 121°C and 1.2 atm) and moistened with distilled sterile water, up to the substrate's saturation level.

The flasks were then exposed to a 16-hour photoperiod, with a luminous intensity of 75 μmol m^-2^ s^-1^ and a temperature of 25±2°C. Of the germinated seedlings, only the ones that had just two sprouted leaves with lengths of 20±2 mm were selected.

### *In vitro* bioassay

The substrate used for the symbiosis test was made of expanded vermiculite (particles of 2.50 to 5.00 mm; density of 110 to 140 kg/m^3^; pH_25⁰C_ = 7.0 to 11.0). Test tubes with dimensions of 150 mm in length and 24 mm in diameter were used to pack 2.3±0.1 grams of vermiculite (Fig 1A). The test tubes were then autoclaved for one hour, at 121°C and 1.2 atm. Subsequently, 14 mL of a modified liquid MNM culture medium was added to each test tube before they were again autoclaved at 121°C and 1.2 atm, for 16 minutes.

Squares of culture medium measuring 1 cm^2^ and containing 0.01 gram of mycelium were selected from the areas closest to the rim of the Petri dish plates used to grow the fungi colonies. One medium-mycelium square was packed in each test tube approximately 1 cm below the surface of the substrate (Fig 1A). The tubes were then kept in a BOD incubator, at 25±1°C, for 15 days, at which time the mycelium covered half of the substrate volume (Fig 1).

The *in vitro* germinated Red Araza seedlings were washed with distilled sterile water, disinfected in a solution of sodium hypochlorite (NaClO, 1.5% active ingredient) for 2 minutes, again washed three times with distilled sterile water, and, finally, kept in a 500 mg L^-1^ solution of sterile polyvinylpyrrolidone (PVP), until they were added to the test tubes.

The seedlings were then treated with the isolates according to the following schema (also represented by Fig 1B): ***Ct***–Control, no fEcM isolate was added to the test tube containing the seedling; ***Sc***–seedling was added to the test tube containing the UFSC–Sc133 isolate; ***Pi*–**seedling was added to the test tube containing isolate D17. After adding the seedlings to the substrate, the base of the test tubes was covered with aluminum foil.

### Analysis of formed ectomycorrhizae

The mycorrhizal colonization index (in %) was calculated according to Brundrett et al. [29] after 35, 70 and 110 days of plant development, and the presence of ectomycorrhizal components, or lack thereof, was determined by microscopic analysis of light and electron scanning of the roots.

In order to anatomical analyze the root cortex, all materials were prepared according to specifications established by Johansen [30], and O'Brien et al. [31], with the following modifications: root samples were fixed in formaldehyde/acetic acid/water (1:1:1 by volume), at 5 ^o^C; then, they were dehydrated through an ethanolic series (60, 70, 80, 90, 96% v/v); and, finally, the samples were infiltrated with historesin (Leica Historesin, Heidelberg, Germany). Cross-section samplings of 5 μm (microtome, Leica RM2125RT) were prepared and stained with a 0.05% solution of O-toluidine blue [31] for a histological analysis.

To scanning electron microscope analyze, the roots samples fixed were dehydrated through an ethanolic series (50, 60, 70, 80, 90, 96% v/v) and dried with HMDS (hexamethyldisilazane, EMS®). After that, the samples were metalized with gold (Leica EM-SCD500) and then observed in ascanning electron microscopy (JEOL JSM-6390LV).

### Plant development analysis

Evaluations were done at 35, 70 and 110 days after the seedlings were added to the test tubes. The relative growth rate (RGR, in mm mm^-1^ day^-1^) and number of leaves completely formed were assessed for each seedling. After sectioning the root's collar region, the aerial part's fresh and dry mass were measured (in mg); the dry mass, specifically, was determined after drying it in an incubator at 65°C until a constant weight was reached, and later measured with a precision scale. Additionally, the length of the root system was measured (in mm), from the main root's collar to its apex.

### Statistical analysis

The experiment was performed in a completely randomized design. Data from the different experiments were submitted to the Shapiro-Wilk normality test and to an analysis of variance (ANOVA), both with *P* < 0.05. The Tukey Test (*P*<0.05) was used to separate the means and determine significant differences. The dry mass and the RGR means did not yield normality (*P* < 0.05), and were transformed by Box-Cox through ((x^-0.104205^)-1)/(0.104205) and ((x^-0.252942^)-1)/(0.252942), respectively. Microsoft Excel® 2013 and R® v. 3.2.3 were the programs used for the analyses.

## Results

### Ectomycorrhizal symbiosis formation

**Fig 2:**
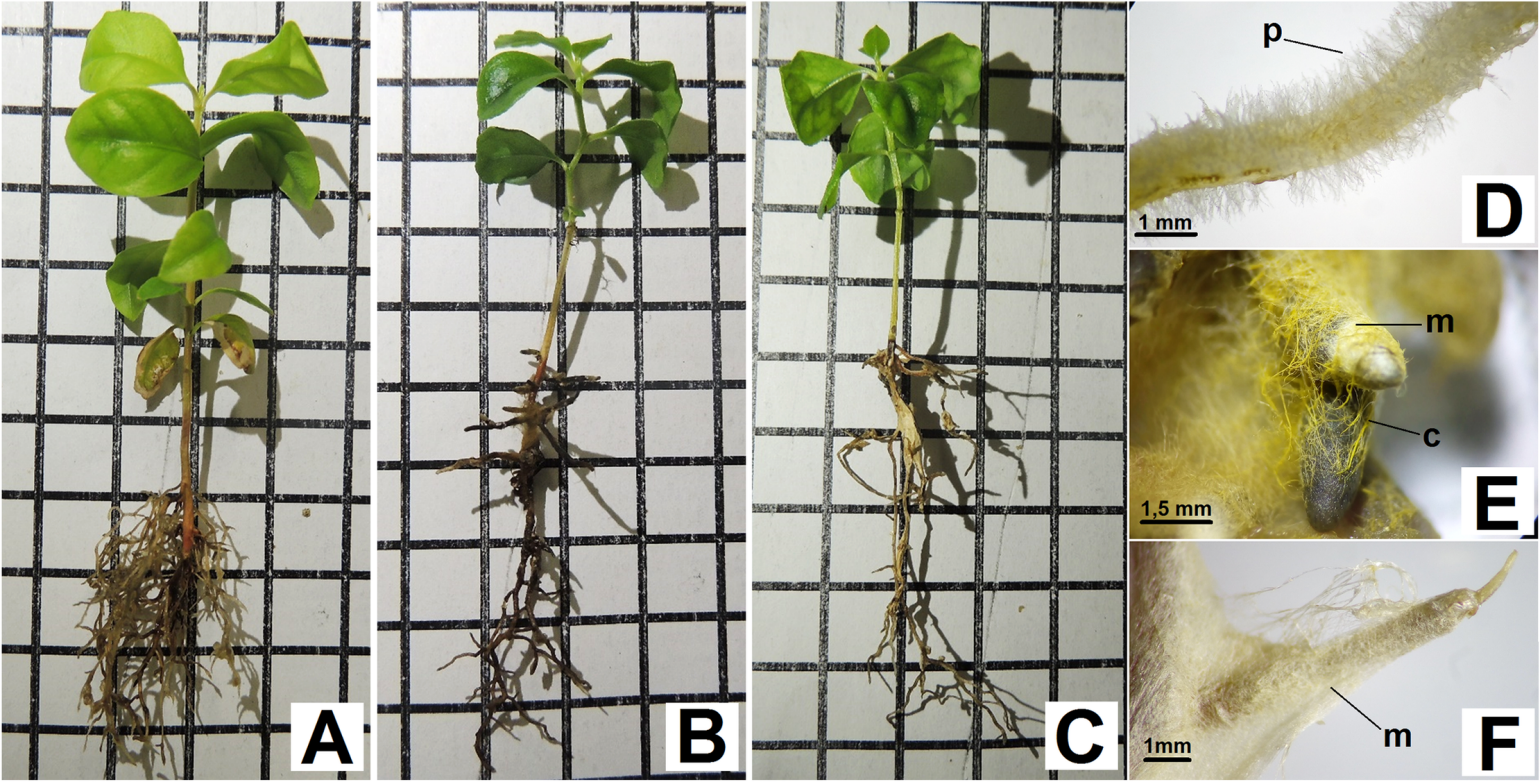
**General aspects (A, B and C) and details (D, E and F) of the root system from the Red Araza plants inoculated with ectomycorrhizal fungi (fEcM), after 110 days of** *in vitro* **cultivation.** (A) and (D) Control plants, no fEcM inoculation. (B) and (E) Plants inoculated with *Pisolithus microcarpus* (D17). (C) and (F) Plants inoculated with *Scleroderma citrinum* (UFSC-Sc133). (p, radicular hairs; m, fungal mantle; c, mycelial cords). Squares were dimensioned at 1 cm by 1 cm.

No interaction was established between the types of fungi and the date in which the mycorrhization was evaluated (*P* = 0.36); only the isolated influence of these individual factors was verified (*P* < 0.01 for both) (Table 1). Mycorrhizal colonization was detected on the Red Araza plants that were inoculated with both tested fungi, D17 and UFSC-Sc133, as can be verified in Table 1 and Fig 2. It is important to note that mycorrhization was not found in the Control plants (Table 1), which suggests that the experiment was not contaminated by other types of ectomycorrhizal fungi.

**Table 1:** Ectomycorrhizal colonization percentage for Red Araza (*Psidium cattleianum*) plants after 110 days.

	Ectomycorrhizal colonization (%)			
Treatment	35^th^ day	70^th^ day	110^th^ day	Average
Control	0.00 ± 0.00	0.00 ± 0.00	0.00 ± 0.00	0.00 C
D17	61.68 ± 24.58	69.42 ± 18.92	91.60 ± 11.64	74.23 A
UFSC-Sc133	1.00 ± 0.57	18.58 ± 9.39	15.70 ± 7.75	11.76 B
**Average**	20.89 b	29.33 ab	35.77 a	

The data are means of at least eight plants ± SD. D17 –*Pisolithus microcarpus*; UFSC-Sc133 –*Scleroderma citrinum*. Means appended by the same letter, lower case within a row and upper case within a column, do not differ statistically from each other according to the Tukey Test (*P* < 0.05).

Regardless of the date of the evaluation, the root systems of the plants inoculated with D17, typically, had a lower number of roots (Fig 2B), but were more robust and showed a thickening of the stem apex (Fig 2E). Moreover, the roots had a darker color (from orange-brown to dark brown) and did not have radicular hairs (Fig 2E), differing in this respect from the Control plants (Fig 2D).

The plants inoculated with the UFSC-Sc133 (Fig 2C) isolate also had root systems with a lower number of roots, when compared to the Control plants; they were, however, completely or partially covered by an expansive, white mycelium coat (Fig 2F), typical of the symbiotic relationship with the *Scleroderma* genus [32]. Only a small part of the root (an average of 11.76%) showed any thickening of the apex or any other morphological alterations, when compared to the root systems of the Control plants.

**Fig 3:**
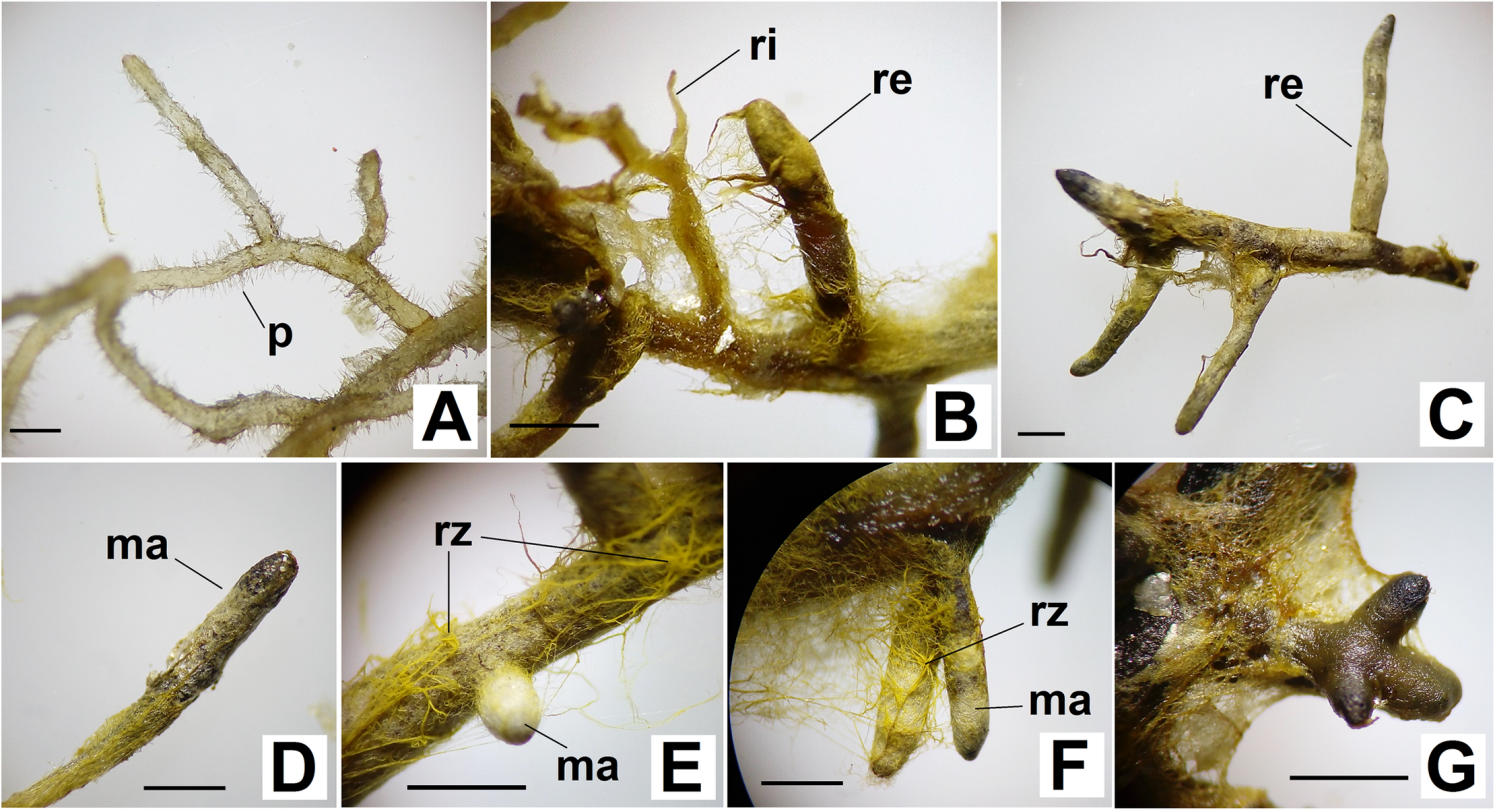
**Root system details of the Red Araza non-inoculated plants (A) and of the Red Araza inoculated *in vitro* with the D17 (***Pisolithus microcarpus***) ectomycorrhizal isolate (B, C, D, E, F and G).** (A) Roots non-colonized by radicular hairs (p). (B) detail showing roots partially (ri) and completely (re)covered by mycelium. (C) Sample showing a colonized root's pinnate monopodial morphotype (re). (D) Secondary root covered by yellowish mycelium, with mantle (ma) formation on the apex. (E) and (F) Secondary roots covered by the mantle, with yellowish rhizomorphs (rz). (G) Details of the dichotomized secondary roots. Bars represent a 1 mm scale.

Most of the root system in the plants with the D17 isolate was covered by an expansive, thin mycelium coat (Fig 3B). Much of the secondary roots were completely covered by this whitish mycelium coat, characterizing a well-developed fungal mantle (Fig 3C, 3E and 3F). In general, the ectomycorrhizae that formed was of the pinnate monopodial morphotype (Fig 3C), rarely dichotomized (Fig 3G). Thicker mycelial cords (rhizomorphs) with a yellowish to yellow-brownish coloration were often observed (Fig 3E and 3F).

**Fig 4:**
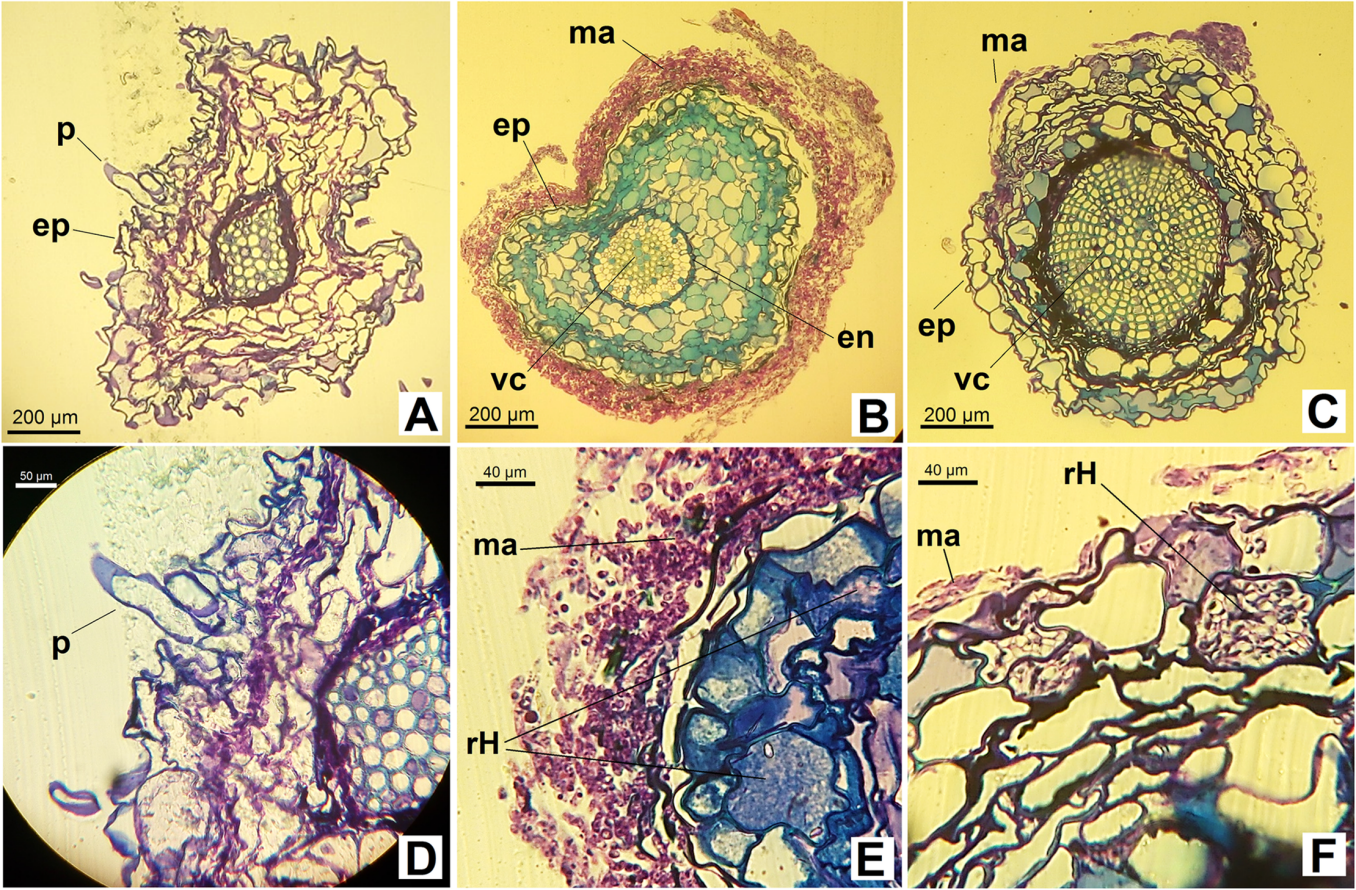
**Root cross-sections of the Red Araza non-inoculated plants (A, D) and of the Red Araza inoculated** *in vitro* **with the D17 (B, E) and UFSC-Sc133 (C, F) isolates.** D17 –*Pisolithus microcarpus*; UFSC-Sc133 –*Scleroderma citrinum*. Note the presence of radicular hairs (p) in A and D, and the formation of a mantle (ma) that partially (C and F) or completely (B and E) covers the root. E and F show the presence of a Hartig net (rH). (ep, epidermis; vc, conducting vessels; en, endoderm).

**Fig 5:**
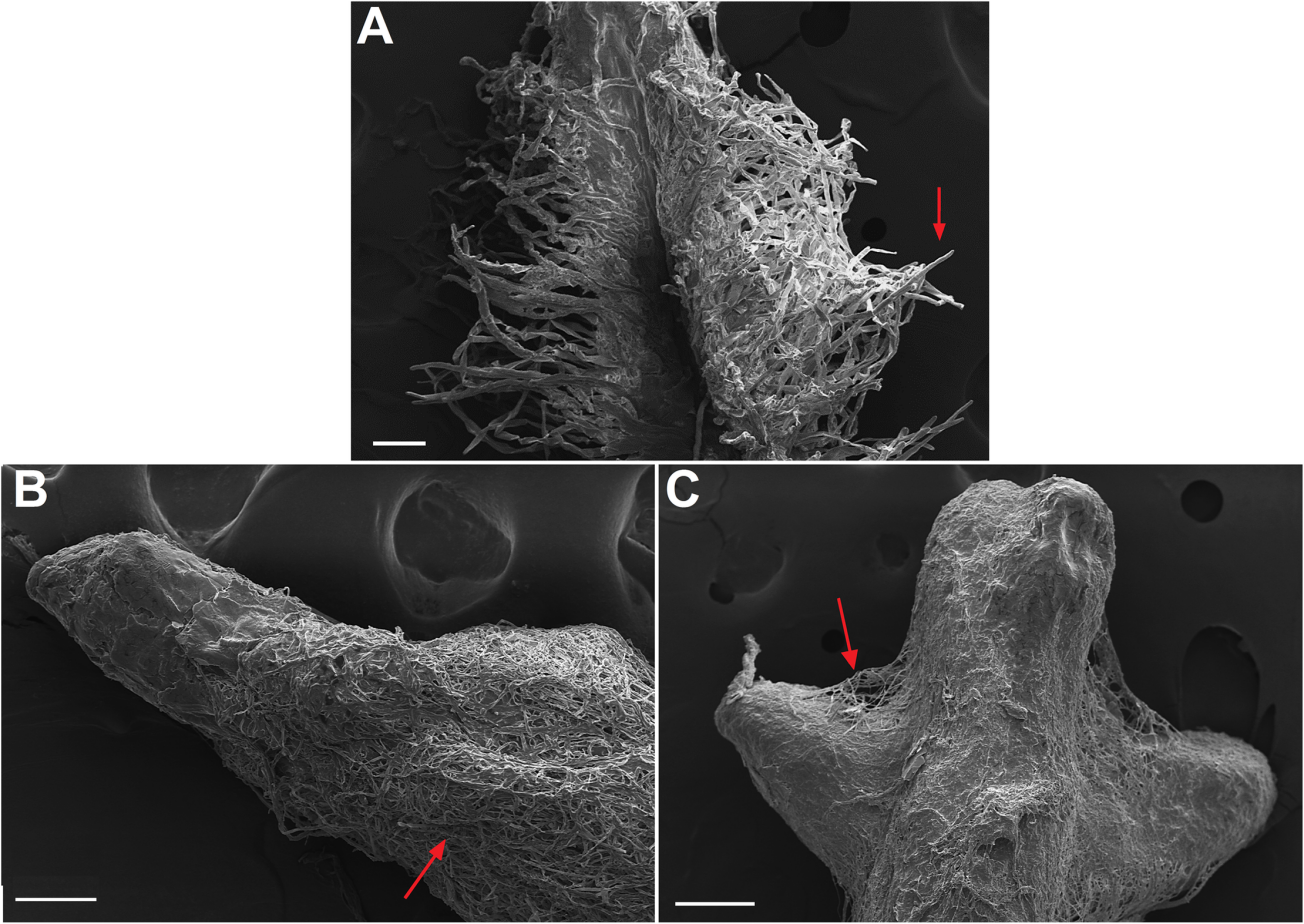
Scaning electron micrograph of Red Araza (*Psidium cattleianum*) roots. (A) Control root, arrow shows root hairs. (B) and (C) Ectomycorrhizae formed by symbiotic relationship with the D17 isolate (*Pisolithus microcarpus*), arrow shows thick fungal mantle. Bars on A and B represent a 100 μm scale and, on C, a 200 μm scale.

The plants inoculated with the D17 fungus exhibited a relatively well-developed mantle around the epidermis (Fig 4B), with little hyphae penetration to the cortex. Although both root thickening and mantle formation did occur (Fig 5B and 5C), microscope views of the cross-sections also revealed the presence of a lightly identifiable Hartig net (Fig 4E). The plants inoculated with the UFSC-Sc133 isolate also exhibited a mantle, though it was underdeveloped and appeared only in some regions of the root system (Fig 4F). A few of the root cells also formed a Hartig net (Fig 4F).

### Plant development analysis

Table 2 contains the plant development data. There was a significant interaction between evaluation dates, and the factors number of leaves (*P* = 0.39) and RGR (*P* < 0.01). The evaluation date and the type of fungus used were significant in terms of the plants' dry matter (*P* < 0.02 and *P* < 0.01, respectively) and their shoot dry mass (*P* < 0.01 for both). In terms of length of the largest root, however, only the evaluation dates were significant (*P* < 0.01).

**Table 2:** Number of leaves, fresh and dry shoot masses, largest root length and relative growth rate (RGR) of Red Araza plants (*Psidium cattleianum*) in different phases of ectomycorrhizal fungi inoculation and without inoculation.

	**Number of leaves/plant**			
**Treatment**	**35**^**th**^ **day**	**70**^**th**^ **day**	**110**^**th**^ **day**	**Average**
Control	3.89 ± 0.42 bA	6.67 ± 0.74 aA	7.89 ± 1.46 aA	6.15
D17	4.87 ± 0.66 aA	6.00 ± 0.75 aA	6.60 ± 1.48 aA	5.82
UFSC-Sc133	2.71 ± 0.82 bA	6.00 ± 1.43 aA	7.33 ± 1.04 aA	5.35
**Average**	3.82	6.22	7.27	
	**Fresh shoot mass (mg)**			
**Treatment**	**35**^**th**^ **day**	**70**^**th**^ **day**	**110**^**th**^ **day**	**Average**
Control	0.394 ± 0.011	0.733 ± 0.031	0.985 ± 0.048	0.704 A
D17	0.283 ± 0.005	0.290 ± 0.005	0.525 ± 0.020	0.366 B
UFSC-Sc133	0.180 ± 0.004	0.249 ± 0.010	0.553 ± 0.012	0.328 B
**Average**	0.286b	0.424b	0.687a	
	**Dry shoot mass (mg)**^1^			
**Treatment**	**35**^**th**^ **day**	**70**^**th**^ **day**	**110**^**th**^ **day**	**Average**
Control	0.009 ± 0.002	0.018 ± 0.009	0.023 ± 0.011	0.017 A
D17	0.008 ± 0.001	0.010 ± 0.002	0.017 ± 0.006	0.011 AB
UFSC-Sc133	0.007 ± 0.002	0.008 ± 0.002	0.012 ± 0.003	0.009 B
**Average**	0.008 b	0.012b	0.018 a	
	**Largest root length (mm)**			
**Treatment**	**35**^**th**^ **day**	**70**^**th**^ **day**	**110**^**th**^ **day**	**Average**
Control	23.33 ± 5.70	29.44 ± 12.40	32.11 ± 9.24	28.29^ns^
D17	18.25 ± 4.63	23.50 ± 5.13	27.00 ± 5.40	22.92^ns^
UFSC-Sc133	14.86 ± 6.37	23.71 ± 4.61	36.56 ± 13.83	25.04^ns^
**Average**	18.81 b	25.55 ab	31.89 a	
	**Relative growth rate (mm mm**^**-1**^ **day**^**-1**^**)**^2^			
**Treatment**	**35**^**th**^ **day**	**70**^**th**^ **day**	**110**^**th**^ **day**	**Average**
Control	0.013 ± 0.006 aA	0.011 ± 0.003 aA	0.009 ± 0.002 aA	0.011
D17	0.017 ± 0.004 aA	0.008 ± 0.002 bA	0.006 ± 0.002 bA	0.010
UFSC-Sc133	0.008 ± 0.006 aB	0.006 ± 0.001 aA	0.006 ± 0.002 aA	0.007
**Average**	0.0127	0.0082	0.0071	

The data are means of at least eight plants ± SD. D17: *Pisolithus microcarpus*; UFSC-Sc133: *Scleroderma citrinum*. For each variable, recorded values appended by the same letter, lower case within a row and upper case within a column, do not differ statistically from each other according to the Tukey test (*P* < 0.05). ^ns^ Not significant.

^1^Original values are shown; for statistical analyses, the values were transformed by ((x^-0.104205^)-1)/(0.104205)

^2^Original values are shown; for statistical analyses, the values were transformed by ((x^-0.252942^)-1)/(0.252942).

In terms of number of leaves, shoot dry mass, root length, and RGR, there were no significant differences between the plants treated with the D17 fungus and the Control group (Table 2). The average fresh mass of the D17 inoculated plants were significantly lower than that of the Control group, which had no fungi (Table 2). Similar data was obtained for the plants inoculated with the UFSC-Sc133 isolate, which also differed from the Control plants in terms of number of leaves, root length, and RGR; additionally, its average for the variables shoot fresh mass and dry mass, were also lower than that of the Control (Table 2).

## Discussion

With the exception of studies done with species of *Eucalyptus*, the existing literature has limited information available regarding the formation of ectomycorrhizae by Myrtaceae native to the Brazilian Atlantic Forests. Research efforts involving species of trees native to this biome show that they are predominantly associated with arbuscular mycorrhizal fungi [33,34]. For the species of the *Psidium* genus, for example, the only information available is related to endomycorrhizal relationships, including efforts involving field [35] and greenhouse studies [36,37] of the guava tree (*P*. *guajava*), as well as of the *P*. *cattleianum* in controlled environment conditions [38].

The present study is the first report in the scientific literature community that involves the symbiotic relationship between Red Araza plants and fEcMs. The ectomycorrhization was confirmed between this Myrtaceae and the D17 (*Pisolithus* microcarpus) and UFSC-Sc133 (*Scleroderma citrinum*) fungi through the formation of a fungal mantle that covered the symbiont's root system, morphological alterations characteristic of that type of relationship, and the formation of a Hartig net (Figs 3, 4 and 5).

It was observed that the D17 isolate produced ectomycorrhizal modifications more quickly and effectively in Red Araza plants than the UFSC-Sc133 isolate. The D17 isolate produced fungal mantle in more than 60% of the root system in all evaluations performed, even under conditions of high concentration of nutrients present in the substrate and stresses caused by the *in vitro* system. This indicates that this isolate has a high compatibility with the Red Araza plants and can be an early colonizer in places with edaphic stresses, as has been observed for other associations between *Pisolithus* and Myrtaceae species [39]. Souza [40] also observed higher colonization of a *Pisolithus* isolate in *Eucalyptus dunnii* seedlings compared to other fungal isolates (including *Scleroderma*), even at high concentrations of phosphorus.

As is the case in this study, species of the *Pisolithus* and *Scleroderma* genera have already been identified in ectomycorrhizal formations with other Myrtaceae present in tropical, subtropical and neotropical ecosystems. *Pisolithus* species, for example, are known to form ectomycorrhizae in *Tristaniopsis guillainii* [41], and in other species of the *Tristaniopsis*, *Melaleuca* and *Sannantha* genera in the ultramafic soils of New Caledonia [42,43]. *Scleroderma* species have been known to form symbiotic relationships with *Gomidesia spectabilis* in dune ecosystems native to Brazil [44]. Four different species of *Scleroderma* have also been identified in neotropical ecosystems native to the Guyanas, dominated by trees of the *Dicymbe*, *Aldina* (Fabaceae) and *Pakaraimaea* (Dipterocarpaceae) genera [7,5].

Many studies show that the establishment of ectomycorrhizal association depends on a complex gene expression/suppression relationship present in both the fungi and the plants [45–47]. It is also well-known that, even before the physical contact between the root and the hyphae, the molecular signals stemming from this genetic interaction are decisive to the compatibility, or lack thereof, between the fungi/plant's ectomycorrhizal symbiosis [48–50]. Thus, the establishment of *in vitro* ectomycorrhizae shows that the Red Araza is capable of producing a few or several of these genetic/molecular interaction mechanisms between it and the ectomycorrhizal fungi [46].

The fact that Red Araza establishes *in vitro* ectomycorrhizae through complex genetic/molecular interaction mechanisms suggests that this same interaction may be occurring in native ecosystems to the Atlantic Forests of Southern Brazil. Researches about the presence of ectomycorrhizae in different ecosystems native of Brazil supports this idea. Examples of this include reports of ectomycorrhizal symbiosis formation in native Myrtaceae to that biome, such as in trees of the *Campomanesia* and *Eugenia* genera [51], and studies that show an *in vitro* association between fEcMs and the grapia (*Apuleia leiocarpa*) and canafistula (*Peltophorum dubium*), species native to Southern Brazil's forests [52]. In addition, a large study carried out on Amazonian Lowland White-sand Forests in Brazil and French Guyana found 62 morphospecies of ectomycorrhizae associated with native trees in these areas [4].

In the present study it was observed the formation of mantle and Hartig network in the ectomycorrhizal association, but did not show an increase in development for the Red Araza plants inoculated with the fEcM in comparison to the Control plants (see Table 2), possibly due to the use of an *in vitro* system. Andreazza *et al*. [52] obtained similar results in their studies, in which, even though a fungal mantle did develop, no difference was detected between the control plants and the *in vitro* fEcM (*Suilus* sp.) inoculated canafistula plants in terms of height, root system fresh mass, and fresh and dry shoot masses.

It was expected that the *in vitro* system would limit the benefits of mycorrhizal associations, due to the high availability of water and nutrients in the substrates [53], a factor that mitigates the positive effects [54, 55]. The *in vitro* system was nevertheless used in this study with the intent of characterizing the Red Araza's actual ectomycorrhizal *status*, without significant environmental variations, such as the presence of other fungal species.

It has, therefore, become evident that further research is necessary to determine if other fEcM isolates can also form ectomycorrhizal associations with the Red Araza plant, and if these relationships are effective in enhancing the plant's development. Finally, further research is also necessary to understand the ectomycorrhizal *status* of other Myrtaceae native to Brazil and, just as it was done for this study involving the Red Araza, find out whether they can form these types of relationships, be it naturally or *in vitro*.

## Conclusion

This study established that *in vitro* D17 (*Pisolithus microcarpus*) and UFSC-Sc133 (*Scleroderma citrinum*) ectomycorrhizal fungi isolates are capable of promoting root system alterations, and forming a fungal mantle and a Hartig net in Red Araza plants, a fact never before registered in the existing scientific literature. The *in vitro* isolate assays did not contribute to an increase in the plants' developmental characteristics when compared to the Control plants, possibly due to the specific experimental conditions used.

Finally, considering that the Red Araza did develop complex molecular relationships by establishing an ectomycorrhizal symbiosis *in vitro*, one can deduce that it, and other Myrtaceae species native to Brazil, can also establish these relationships in its/their natural ecosystems.
